# Treatment of Fractures

**Published:** 1862-01

**Authors:** 


Treatment of Fractures.—There is no one thing probably which better exemplifies the "progress of surgery," than the present management of fractures. With what uncouth lumbersome machines the unfortunate patients used to be encased, and how bed-ridden they got, whereas now the dressing is so light, strong, and safe, that the man with a broken leg walks in the garden. Our New York surgeons, seem not yet all to appreciate this style. They revere antiquities; and I will venture to say that there are more of the old clumsy fracture apparatus, now used in the three New York Hospitals, than in all of the sixty-two like institutions in Paris.

The modern substitutes for *planks*, etc., are of course familiar to you. Dextrine, gelatine, caoutchouc, plaster of Paris, are all in vogue. The last mentioned is evidently the best, for it has been under trial since the time of the elder Larrey, who, it is said, first used it; and to this day it stands the most popular. The methods of application have naturally changed as experiences accumulated. Larrey, for instance, moulded the limb in it—others put on a satureted circular bandage—others again dusted the powdered plaster over wet bandages—others still applied it with a trowel as on a wall; and so on to the end, or rather until the arrival of Maisonneuve. The question is now settled, and all other modes are obsolete. M. Maisonneuve had once the good luck to receive a fracture of the leg, and so this method he first planned on his own person.

For example and explanation, let us suppose a broken tibia. He orders four coarse cloths, longer than the leg, and each so broad that when folded three or four times, they will be respectively reduced to a breadth of from two to four inches—the breadth, of course, varying with the limb under treatment. After these cloths have thoroughly soaked in the fluid plaster, they are taken dripping from the vessel, and directly applied along the sides of the limb. The lower ends of three of them embrace the foot and lap intimately on the sole. The front one passes on the dorsum to the toes. A circular bandage, saturated or not, is then quickly rolled up to the knee, and perhaps down again to the foot. When this bandage has not been soaked in the plaster, the apparatus is called *demi-platre.* In five minutes after everything is solid. The liquid splints have become firm, and every point is supported with an impartiality heretofore unapproached.

Unless when the soft parts are greatly injured, M. Maisonneuve often applies this apparatus immediately, or may be one day after the accident. This seems unsafe, and indeed I have sometimes seen most discreditable results. However, if any trouble is suspected of existing, there is no apparatus, as a permanent one, that can afford a more easy approach to the limb. And the debarrassment need be only *partial;* for after the removal of the bandage one splint, two splints, or only part of a splint, can be readily taken off, and the remainder stands firmly to its duty. If the parts thus exposed require washes, such can be used with impunity. The splints are not easily softened. In like manner are treated almost all fractures—femur, patella, clavicle, etc., etc.

A great many have failed to have success with this procédé of Maisonneuve, and it has been often because the cloth used was too *fine.* Some coarse material is the thing, such as old coarse hospital sheeting. The solution of plaster should be as thin almost as milk.—*Cor. Am. Medical Times.*

